# Cognitive behavioral group intervention for pain and well-being in children with juvenile idiopathic arthritis: a study of feasibility and preliminary efficacy

**DOI:** 10.1186/s12969-015-0032-x

**Published:** 2015-08-21

**Authors:** Johanne Jeppesen Lomholt, Mikael Thastum, Anne Estmann Christensen, Anne Leegaard, Troels Herlin

**Affiliations:** Department of Psychology and Behavioural Sciences, Aarhus University, Bartholins Allé 9, 8000 Aarhus, Denmark; Department of Pediatrics, Odense University Hospital, Sdr. Boulevard 29, 5000 Odense C, Denmark; Department of Pediatrics, Aarhus University Hospital, Brendstrupgaardsvej 100, 8200 Aarhus N, Denmark

**Keywords:** Feasibility, Juvenile idiopathic arthritis, Cognitive behavioral therapy, Pain, Functional disability, Health-related quality of life, Pain cognitions

## Abstract

**Background:**

Pain is still a part of everyday living for several children with juvenile idiopathic arthritis (JIA) despite improvement in treatment. Psychological interventions may contribute to diminish pain complaints and improve well-being in children with JIA. Only few studies have investigated the efficacy of psychological therapy in children with arthritis and with mixed results.

The aim of the study was to evaluate the feasibility and preliminary efficacy of a cognitive behavioral therapy group intervention for children with JIA and their parents.

**Methods:**

Nineteen children with JIA and their parents were allocated to six sessions’ group cognitive-behavioral therapy (n = 9) or a waitlist control condition (n = 10). Results were measured from self-reported scales and one-week pain diaries. Clinical data was collected by a rheumatologist.

**Results:**

The participation rate was low; 33 % of the invited families participated. However, the participants rated the intervention’s credibility and satisfaction with the intervention as high. The dropout rate was low and attendance rate high. Increased quality of life and improvements in adaptive pain cognitions was reported in the intervention condition compared to the waitlist condition, whereas no differences were found for pain and functional disability. The disease activity increased in the treatment condition but not in the control condition.

**Conclusions:**

The feasibility of this study seemed high with regards to the acceptability of the families participating in the intervention. However, the feasibility in general was challenged by implementation problems with a low participation rate. A reduction in pain after the intervention was not found even though pain management was the main target in the intervention. Preliminary analysis showed that although the severity of the disease status increased, an increase in quality of life, reduction in pain catastrophizing, and an improvement in adaptive pain cognitions (the beliefs in controlling pain and self-efficacy) were seen in the intervention condition. The study highlights the importance of considering the disease status when evaluating the efficacy of a psychological intervention in children with arthritis. Conclusions on the strength of the efficacy require further research in a large, randomized controlled trial.

## Background

Juvenile idiopathic arthritis (JIA) is the most common childhood rheumatic disease and the incidence rate of JIA in Scandinavian countries is reported to be 14–15 per 100 thousand children per year [1, 2]. JIA comprises seven categories that have in common a chronic arthritis with no other explanation. In addition, common for all categories of JIA is pain, morning stiffness, and loss of function caused by the arthritis [3]. On average, children with polyarticular JIA reported pain on 73 % of the days over a two-month period [4], and in another study 29 % of JIA patients who achieved remission on biologics reported daily pain in a two-week period [5]. For a child with JIA the amount of pain experienced is modulated by psychological, social, and biological factors, independently of the inflammatory disease activity [6].

Pain cognitions are associated with pain experience, but the causality is still unclear. However, changes in the pain beliefs of control (belief of having more control over pain), disability (belief of being functionally impaired), and harm (belief that pain signifies damage), pain catastrophizing, and self-efficacy for managing pain, mediated the effects of cognitive behavioral therapy (CBT) on pain and functional disability in adult patients with chronic pain [7]. All pain cognitions were significantly associated with pain intensity in samples of children with JIA [8, 9].

Modifying mal-adaptive pain cognitions is the purpose of CBT and studies in pediatric populations with chronic and recurrent pain have shown promising results in pain reduction using CBT [10, 11].

The few studies examining the efficacy of psychological intervention in children with arthritis have produced diverting results with regards to pain, functional disability, and health-related quality of life (HRQL) [12–14]. In one randomized, waitlist-controlled study by Lavigne et al., there were no differences in pain reduction between the intervention and control group [12]; conversely, another study by Walco and colleagues without a control group found significant reductions in pain [13]. Both studies were hampered by small sample sizes (8 and 13, respectively). A more recent pilot study by Stinson et al. with a randomized controlled design investigated the effect of a web-based, self-management intervention program for 48 adolescents with JIA. The study found significant improvements in pain scores with a large effect size (Cohen’s d = 0.78). The participants did not report an improvement in either functional disability or psychosocial dimensions in their HRQL [14].

Studies have found that the severity of the disease activity in children with arthritis predicted a small to medium part of the variance in pain intensity reported by the children [15–18]. The disease activity in JIA can be unpredictable and changing in some children, which may influence the child’s pain experience and affect the efficacy of a psychological intervention. In the previous psychological intervention studies with children with arthritis outlined above the disease status was not included and the impact of possible changes in disease status was not addressed.

The current study uses common CBT techniques for pain management but differs from previous psychological interventions in children with arthritis by including parents in the therapy and by delivering the intervention in a group format. The inclusion of a family component in the CBT treatment has shown to be effective in reducing chronic pain [19] but studies comparing individual CBT to group CBT in chronic pain patients have found no differences in efficacy [20–23]. Considering practitioner time and patient expenses, group therapy may be more cost effective than individual therapy.

Designed as a waitlist-controlled trial the aim of the study was to develop and evaluate the feasibility and preliminary efficacy of a psychological group intervention program based on CBT-principles for children with JIA and their parents. Firstly, we hypothesized that the intervention would show evidence of feasibility defined as acceptability as demonstrated by high-attendance and low drop-out rate, parent’s belief in the credibility of the intervention early in the course, and the participant’s satisfaction with the intervention after completion. Secondly, we hypothesized that, after controlling for differences in disease status, the intervention condition would show a reduction in symptoms (pain intensity and level of functional disability), an improvement in HRQL, and a modification of maladaptive pain cognitions compared to the control condition.

## Methods

### Setting and participants

During routine visits within a 5 months period at two Danish Pediatric Rheumatology Clinics all children (n = 129) with JIA, aged 9–14 years, assessed their average pain during the week on a Revised Faces Pain Scale (FPS-R) [24]. Inclusion criteria for the trial were a confirmed JIA diagnosis according to the International League Against Rheumatism (ILAR) criteria [25]; lack of comorbidity with other chronic diseases; ability to speak fluent Danish; and a pain assessment above the median for the total sample (≥2.0). The inclusion criteria based on the child’s pain score were selected to make the intervention and exercises with pain-management relevant for the participating children. This study was approved by the Danish Data Protection Agency and the Ethical Review Board, Central Denmark Region, Denmark, and the recruitment procedure was based on their guidelines.

### Procedure

**Fig. 1:**
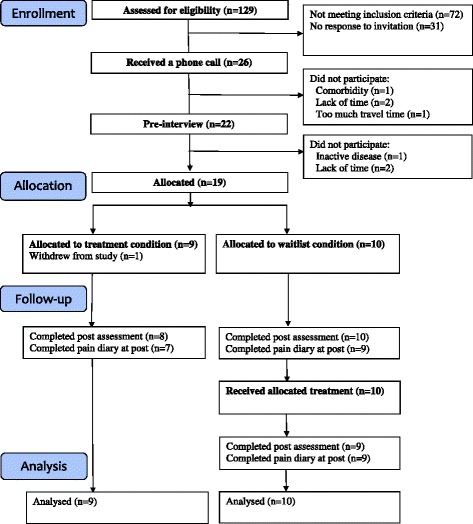
A flow diagram of the study

Fifty-seven children and their parents were eligible for inclusion and invited. Twenty-six families who responded positively to the invitation received a phone call with information about the study. Of these, 22 agreed to participate in a pre-interview where written consent was obtained and finally 19 children (15 girls and 4 boys, mean age = 11.7 years, SD = 1.7), and their parents participated in the intervention. Figure 1 represents a flow diagram of the study.

The intervention was conducted in groups and took place in three different cities in Western Denmark. To reduce travelling time for the participating families, they were assigned to a group geographically closest to their home address. In one of the groups more than six families were allocated. Therefore, the group was divided into two and the allocation of the families was based on the children’s age to obtain groups with children of almost the same age. This resulted in 4 groups, which were randomly allocated into either an intervention or a three-month waitlist condition. Families at the waitlist-condition were offered treatment after the waitlist period.

Children and parents in the intervention condition completed questionnaires before (at the pre-interview) and after the 8 weeks therapy. The child completed a daily pain diary the week following completion of the questionnaires. Families on the waitlist completed questionnaires and pain diaries at three occasions: before the three-month waitlist period (at the pre-interview), before therapy, and after therapy. All participating parents completed a credibility measure after completion of session I. Except for the questionnaires completed at the pre-interview; all questionnaires were web-based and emailed to the families to be completed at their home. The children were examined by a pediatric rheumatologist when they came to the clinic for regular, routine visits, and the disease activity was registered. An informed consent was signed by the parents.

### Psychological intervention

The intervention consisted of six weekly sessions lasting 120 min. (The last two sessions were conducted biweekly). The two psychologists (JJL and MT) involved in the intervention were trained in CBT, and both were involved in the development of the intervention protocol. Two research assistants with a bachelor’s degree in psychology assisted during the sessions. To maintain intervention fidelity, intervention content and progress were discussed continuously between the psychologists and assistants (e.g., in supervision). The intervention was manualized, and both children and parents received a workbook, worksheets, and guides for home practice during the sessions (please contact the corresponding author for electronic versions of the workbooks). The inspiration to the structure of the intervention was the Cool Kids Program [26], which is an evidence based CBT program for children with anxiety disorders. The Cool Kids Program has recently been evaluated in a Danish population with success [27].

The intervention in this study was a manualized group CBT program focusing on psycho-educating children and parents on pain mechanisms, teaching children to restructure pain related negative automatic thinking and gradually confront pain related avoided situations. Each session had the following structure: Homework from the previous session was evaluated, and the theme of the current day’s session was introduced to both parents and children. Parents and children were then separated. While the therapists worked with the children, the parents were given topics to discuss regarding their homework from last session and for the current session. About halfway through the session, the therapists worked with the parents, while the children were having a break. At the end of the session, the parents and children were introduced to the current session’s homework.

In session I, participants were psycho-educated about the gate-control theory and the bio-psycho-social model of pain. When cognitive principles were introduced, the main objective was to clarify the association between thoughts, feelings, behavior, and pain intensity. Both parents and children were encouraged to set treatment goals for themselves and their family. In session II, the focus was cognitive restructuring. In session III, the children continued to work with cognitive restructuring, and they were also introduced to rewards. For the parents, the theme was parenting a child with arthritis. Principles of parent management were introduced with focus on the challenges of being a parent and a family of a child with a chronic disease. In session IV, a distraction exercise was introduced as a method for remaining present in the moment, instead of focusing on worries and pain. Exposure was introduced as steps in a ladder to demonstrate that a challenge can be overcome by dividing it into smaller steps. In session V, social skills and assertiveness training were introduced and the principles were rehearsed in role-play exercises. In the final session VI, strategies that could be used in painful situations were introduced, and the family’s goals from the first session were evaluated.

## Measures

### Feasibility measures

#### The credibility of the intervention

The parents completed a questionnaire about their belief of the intervention’s credibility after the completion of session I. The questionnaire consisted of five items (the items are presented in Table 2), rated on a five point Likert scale (range = 1(“not at all”) - 5(“a lot”)).

#### Satisfaction with the intervention

Both children and parents evaluated their satisfaction with the intervention by completing a modified version of the Experience of Service Questionnaire (ESQ) [28] after the last session. Both children and parent versions included ratings on a three-point Likert scale (range = −1 to 1). A sum score was calculated with a range from −10 to 10 (children’s version) and from −12 to 12 (parent’s version); higher scores indicated higher satisfaction with the intervention.

### Primary outcome measures assessed by children

#### Pain intensity

Pain intensity was measured with the Revised Faces Pain Scale (FPS-R) with anchors that represented 0 = “no pain” to 10 = “worst pain” [24]. The children were instructed to measure their current pain intensity daily every morning and evening over the following week.

#### Functional disability

The children assessed their level of functional disability with the Functional Disability Inventory (FDI), a global measure of functional disability [29]. The inventory consisted of 15 items, each rated on a five point Likert scale (range 0–4); individual item scores were summed to create a total score; higher scores indicated greater pain-related disability. The Cronbach’s alpha internal consistency reliability coefficients were in an acceptable range at all assessments (0.83–0.93).

#### Health-related quality of life (HRQL)

Children assessed their HRQL with the Pediatric Quality of Life Inventory (PedsQL™). The PedsQL™ -4.0 generic core scale [30] consisted of a total score based on 23 items assessed on a five-point Likert scale. The PedsQL™ 3.0 arthritis module [31] consisted of 22 items, and was specifically designed to assess the HRQL of children with arthritis. The three subscales included were treatment, worry, and communication. It is not recommendable to calculate a total score for the PedsQL™ 3.0 arthritis module, and therefore the subscales were analyzed separately. For both questionnaires raw scores on the remaining scales were transformed to a score of 0 to 100; higher scores indicated higher levels of HRQL. The Cronbach’s alpha was in an acceptable range for all scales (0.70–0.89).

### Secondary outcome measures assessed by children

#### Pain catastrophizing

Level of catastrophizing was assessed on the internalizing/catastrophizing subscale from the Pain Coping Questionnaire (PCQ), which consisted of five items [32]. On a five-point Likert scale (range 1–5), the children indicated how often they were experiencing catastrophizing thoughts when in pain. The Cronbach’s alpha was acceptable (0.73–0.84).

#### Pain-specific beliefs

Pain-specific beliefs was assessed with a revised version of the Survey of Pain Attitudes (SOPA, children’s version) [9]. On a three-point Likert scale (range 1–3), the children indicated how much they agreed with 24 statements about their pain. The statements comprised the three subscales: control (belief in one’s personal control over pain), disability (belief that one is unable to function due to pain), harm (belief that pain signifies damage and that exercise and activity should be restricted). The Cronbach’s alpha for each of the subscales were in an acceptable range (0.63–0.77).

#### Self-efficacy

Disease related self-efficacy was assessed with the Children’s Arthritis Self-Efficacy Scale (CASE) [8]. The children were asked to rate on a five-point Likert scale (range 1–5) how certain they were of being able to manage 11 physical and psychosocial issues related to their disease. These 11 items measured three aspects of the child’s self-efficacy - the “symptoms subscale”, the “emotions subscale” and the “activity subscale”. The Cronbach’s alpha for each of the subscales were in an acceptable range (0.79–0.84).

### Disease measure

#### Disease activity

A composite arthritis activity score (range 0–9) was calculated based on data registered by the pediatric rheumatologists. This score represented an active joint score, the morning stiffness period, and the erythrocyte sedimentation rate [9, 33]. Higher score indicated higher disease activity.

### Statistical analysis

All statistical analyses were performed with IBM SPSS statistics 20.0 for Windows (IBM® SPSS®, IBM Corp., Armonk, New York). Descriptive statistics, including means, *SD*, and frequencies, were computed to report the feasibility outcome and children’s characteristics at admission. Comparisons were based on the chi-square test for categorical variables and the Mann–Whitney *U* Test for continuous variables. Exact *p*-values are presented for each relevant test to facilitate a critical interpretation of the data. In analyses regarding the intervention’s feasibility all participants from both conditions were included.

The two conditions (intervention and waitlist, respectively) were compared at pre- and post-intervention. The results presented are based on intent-to-treat (ITT) analyses. For dropouts in the analyses, pre-intervention scores were moved forward to subsequent assessments, and missing pre-intervention data were replaced with the group pre-intervention mean. In order to maximize the statistical power with this small sample, and to check for differences in pre-scores and disease status, which could affect the outcome, the post scores of the two conditions were compared with an analysis of covariance (ANCOVA); pre-intervention data and differences in disease status (calculated as the difference in disease activity score from pre- to post-intervention) were considered covariates. The exact *p*-value and effect size is reported to detect the magnitude of a potential effect. The partial eta-squared value was used as a measure of effect size, categorized as follows: 0.01 = small effect, 0.06 = medium effect, and 0.14 = large effect [34].

## Results

### Characteristic of the sample

The 19 children that participated in the study did not differ from the non-participating children that met the inclusion criteria in regards to age, gender-ratio, disease duration, pain scores, and disease status (data not shown). Demographic characteristics of the participating children are shown in Table 1. No significant differences were found between the two test conditions regarding demographic characteristics.

**Table 1:** Demographic characteristics of the study population

	Treatment condition (n = 9)	Waitlist Condition (n = 10)	Group differences	*p*-value
Age (year): mean (*SD*)	11.4 (2.0)	12.0 (1.4)	*Z* = −0.82	0.41
Gender (female)	8 (89 %)	7 (70 %)	*χ* ^2^(1) = 0.20	0.66
JIA categories:			*χ* ^2^(6) = 6.83	0.34
Systemic arthritis	1 (11 %)	1 (10 %)		
Oligoarthritis persistent	2 (22 %)	4 (40 %)		
Oligoarthritis extended	2 (22 %)	0 (0 %)		
Polyarthritis (RF-negative)	2 (22 %)	3 (30 %)		
Polyarthritis (RF-positive)	0 (0 %)	1 (10 %)		
Psoriatic arthritis	2 (22 %)	0 (0 %)		
Enthesitis-related arthritis	0 (0 %)	1 (10 %)		
Time from onset to diagnosis (year): mean(*SD*)^a^	0.7 (0.8)	0.8 (0.7)	Z = −0.53	0.60
Disease duration(year): mean(*SD*)^b^	5.1 (4.0)	7.6 (4.0)	*Z* = −1.47	0.14
Current use of medication:				
Methotrexate use	4 (44 %)	3 (30 %)	*χ* ^2^(1) = 0.43	0.52
Etanercept use	3 (33 %)	4 (40 %)	*χ* ^2^(1) = 0.09	0.76
Treatment time with methotrexate (year): mean(*SD*)	0.9 (1.4)	0.9 (1.3)	Z = −0.05	0.97
Treatment time with etanercept (year): mean(*SD*)	0.5 (1.0)	1.4 (2.9)	Z = −2.01	0.91

^a^ Time from onset of symptoms to time of JIA diagnosis

^b^ Time from onset of symptoms to the participation in the study

The children in the intervention condition experienced a small, but insignificant increase in disease activity during the intervention (pre: mean = 0.0, SD = 0.0, post: mean = 0.78, SD = 1.09, *p* = 0.07). In the waitlist condition the children experienced a small, but insignificant decrease in disease activity during the waitlist time (pre: mean = 0.80, SD = 1.60, post: mean = 0.50, SD = 0.97, *p* = 0.28). The disease activity did not change for the children in the waitlist condition during the intervention (post-intervention score: mean = 0.50, *SD* =0.70).

### Descriptive feasibility data

Of the targeted families 19 out of 57 (33 %) participated in the intervention. Of the 18 families, who completed the intervention, 12 families (67 %) participated in all 6 sessions, 4 families (22 %) missed one session, and 2 families (11 %) missed two sessions. A summary of the parent’s ratings of the credibility of the intervention and the family’s satisfaction with the intervention is shown in Table 2. The parent’s average rating of the credibility of the intervention was 4.14 (*SD* = 0.51, range 1–5), and both children and parents indicated a high level of satisfaction with the intervention (Children: mean = 7.76 (SD = 2.36), range −10- 10. Parents: mean = 9.56 (SD = 1.56), range −12- 12).

**Table 2:** Demographic data about feasibility of the intervention

Credibility of the intervention (range 1–5):	Mean (*SD*)
1) At this point, how relevant do you think this intervention was to you?	3.94 (0.70)
2) At this point, how confident are you that this intervention will be rewarding for you?	4.08 (0.75)
3) At this point, how successful do you think this intervention will be in helping other children with arthritis to manage their disease and pain more effectively?	4.19 (0.75)
4) At this point, how successful do you think this intervention will be in helping other parents with children with arthritis manage their child’s disease and pain more effectively?	4.03 (0.76)
5) At this point, would you recommend to others that they should participate in this intervention?	4.47 (0.67)
Total score	4.14 (0.51)
Satisfaction with the intervention	Mean (*SD*)
ESQ parent’s-version (range −12-12)	9.56 (1.56)
ESQ children’s-version (range −10-10)	7.76 (2.36)

### Efficacy analyses

No differences between the intervention condition and the waitlist condition were found regarding pain intensity and functional disability. A difference between conditions was found, for the total HRQL scale (*p* = 0.18) and pain catastrophizing (*p* = 0.21); pain catastrophizing was lower and the HRQL was higher in the intervention condition compared to the waitlist condition. Effect sizes were moderate (range 0.10–0.12). For arthritis-specific HRQL scales, the children in the intervention condition reported a better HRQL, on the worry and communication scales, compared to the waitlist condition with large effect sizes for both scales (range 0.21–0.22) and a *p*-value =0.06. Differences with large effect sizes (range 0.17–0.22) and *p*-values between 0.06 and 0.10 were also found for the SOPA-control subscale and all the CASE subscales. The children in the intervention condition reported a higher belief of being able to control pain and higher self-efficacy than those in the waitlist condition. No differences were found between conditions for the other SOPA subscales (Tables 3 and 4).

**Table 3:** Differences in post-scores in primary outcomes between the condition receiving immediately treatment (n = 9) and the waitlist (n = 10)

Dependent variable		Pre-score		Post-score				Comparison between conditions		
Measure	Range	Mean (SD)		Unadjusted mean (SD)		Adjusted mean (SE)^a^		F-value	*p*-value	Partial Eta^2^
		Treatment	Waitlist	Treatment	Waitlist	Treatment	Waitlist			
*Symptoms:*										
Pain intensity	0–10	3.06 (2.69)	2.70 (1.57)	4.08 (2.66)	2.70 (1.77)	3.50 (0.73)	3.23 (0.69)	0.06	0.81	0.004
Functional disability	0–60	11.37 (3.98)	9.78 (7.91)	11.67 (9.51)	9.20 (9.67)	9.78 (3.12)	10.90 (3.01)	0.06	0.81	0.004
*HRQL:*										
Total (generic)	0–100	62.77 (14.28)	75.95 (14.77)	69.85 (14.05)	72.84 (15.39)	77.63 (4.32)	68.62 (4.03)	1.96	0.18	0.12
Treatment	0–100	73.02 (27.38)	78.73 (15.81)	75.79 (16.92)	80.00 (13.59)	77.99 (4.84)	78.02 (4.55)	0.001	0.98	0.0001
Worry	0–100	70.37 (30.93)	75.00 (19.24)	83.33 (15.02)	77.50 (18.45)	88.37 (5.12)	72.96 (4.82)	4.22	0.06	0.22
Communication	0–100	68.52 (28.19)	61.11 (25.15)	81.48 (14.89)	63.33 (30.48)	85.70 (8.93)	59.53 (8.44)	3.99	0.06	0.21

^a^ Mean and standard error adjusted for covariates (pre-score and differences in disease activity)

**Table 4:** Differences in post-scores in secondary outcomes between the condition receiving immediately treatment (n = 9) and the waitlist (n = 10)

Dependent variable		Pre-score		Post-score				Comparison between conditions		
Measure	Range	Mean (SD)		Unadjusted mean (SD)		Adjusted mean (SE)^a^		F-value	*p*-value	Partial Eta^2^
		Treatment	Waitlist	Treatment	Waitlist	Treatment	Waitlist			
*Pain cognitions:*										
Catastrophizing	1–5	2.24 (0.91)	2.18 (0.57)	1.71 (0.50)	2.02 (0.87)	1.61 (0.27)	2.11 (0.25)	1.67	0.21	0.10
Control beliefs	1–3	1.93 (0.32)	1.76 (0.40)	2.22 (0.41)	1.73 (0.41)	2.22 (0.16)	1.73 (0.15)	4.27	0.06	0.22
Harm beliefs	1–3	2.29 (0.35)	2.05 (0.43)	2.10 (0.29)	2.03 (0.42)	2.01 (0.12)	2.11 (0.11)	0.29	0.60	0.02
Disability beliefs	1–3	2.15 (0.25)	1.68 (0.45)	1.93 (0.40)	1.86 (0.44)	1.76 (0.15)	2.00 (0.14)	1.06	0.32	0.07
Self-efficacy: Symptom	1–5	2.83 (1.06)	2.58 (0.86)	2.97 (0.91)	2.13 (0.82)	2.95 (0.34)	2.14 (0.32)	3.21	0.09	0.18
Self-efficacy: Activity	1–5	2.72 (1.05)	2.67 (1.22)	3.36 (0.53)	2.63 (1.16)	3.40 (0.32)	2.59 (0.30)	3.04	0.10	0.17
Self-efficacy: Emotion	1–5	3.07 (1.22)	2.48 (1.18)	3.37 (1.14)	2.27 (0.95)	3.25 (0.31)	2.38 (0.29)	3.75	0.07	0.20

^a^Mean and standard error adjusted for covariates (pre-score and differences in disease activity)

## Discussion

This study examined the feasibility and preliminary efficacy of a group-based CBT program for children with JIA and their parents. The feasibility of the intervention was investigated focusing on the intervention’s acceptability, compliance to the intervention, and implementation. The acceptability of the study seemed high for the families participating in the intervention. Parents reported high levels of intervention credibility, which are related to motivation for engaging in the intervention [35]. Satisfaction with the intervention was high for both children and parents. Overall, the drop-out rate was low; only one family did not complete the intervention program. Based on all the participating families’ evaluation of the intervention’s acceptability, families who were able and willing to participate in the program found it helpful, useful, and recommendable.

The majority of families attended all sessions, and only two families missed more than one session. The response rate to the assessments was not complete, but, considering the comprehensive assessment (multiple questions in several questionnaires and a one-week diary), a complete response rate may have be difficult to obtain.

One core element in CBT was the homework of the participants between sessions. Information regarding the amount of homework done by each participant was not collected systematically throughout the intervention; however, based on a qualitative estimate half of the children and the majority of the parent’s did not spend time on homework between sessions. The impact of homework on the outcome of CBT interventions has been investigated in a few studies with a pediatric population, but, with inconsistent findings [36–39]. Participant’s low compliance to the homework may have negatively affected the implementation of the CBT strategies in the participants’ everyday life and the efficacy of the intervention. However, based on previous research the benefits of homework in pediatric populations is not well-established, and therefore, the consequences of the low compliance regarding homework is unclear.

The implementation of the intervention showed some problems. Even though we tried to minimize transportation time by doing the intervention at satellite locations, only one third of the eligible and invited families, wished to participate, which may question the relevance of the intervention for the majority of the families we hypothesized could be included. Although this design made randomization impossible at the individual level, it was chosen to minimize the cost for the participating families regarding travelling time and expenses, and, thereby, in hope to maximize the participation rate. Based on qualitative evaluation of the intervention in the last session, an increase in distance to the sessions would have been a reason for not participating in the intervention for some families.

There may be several reasons for the relatively low interest in the study. We had decided to invite families with children with a pain score above the median of the total sample in the clinic. The median pain score, however, was rather low (2 of a range of 0–10). The intervention may therefore have been perceived as irrelevant for some of the invited families. Furthermore, the clinical importance of a reduction in pain in children experiencing a low pain level may be very little, as well as challenging to obtain statistically significant reduction in a low pre pain score. Although the participating sample was similar to the invited sample in disease characteristics, there may have been differences in the socio-economic status between the participating and non- participating sample. It is possible that participants in the rather time-consuming intervention were families with more resources than the non-participants. However, due to the Danish ethical approval system we were not allowed to contact non-participants for more information. Seventy-two patients were excluded due to no pain or very low pain. This criterion was selected to only include children with a present pain problem. Considering the fluctuation in pain in children with arthritis an inclusion criteria based on the pain score may not be recommendable for future studies. Broader inclusions criteria and self-selection from the families may be a better way of reaching families in need of treatment.

The preliminary explorations of the efficacy of this intervention yielded mixed results. Contrary to our hypothesis, no pre-post differences in pain or functional disability were found between the two conditions. However, according to Kashikar-Zuck et al. [40] the functional disability levels in the present sample could be categorized as none or minimal both before and after the intervention. Considering these low levels, a reduction in functional disability may be unrealistic, and not clinically important.

The two previous studies from 1992 found inconsistent results regarding pain reductions [12, 13]. Walco et al. found a decrease in reported pain in an uncontrolled study [12], whereas Lavigne et al. did not found a difference between an immediately treatment group and a waitlist group in regards to mean pain ratings [13]. In both studies the intervention was based on behavioral therapy with focus on muscle relaxation and biofeedback. Since 1992 the diagnosis as well as treatment options have changed substantially regarding children with arthritis. The term JIA was decided in the late nineties [41], and the advent of biological anti-tumor necrosis factor (anti-TNF) agents have improved the treatment options for JIA markedly [42]. The differences in therapeutic techniques, pharmacological treatment as well as sample characteristics make it difficult to compare the results of these studies with our study.

In a more recent study by Stinson et al. with adolescents with arthritis a reduction in pain was found. The intervention was delivered through the internet combined with telephone support in a 12-weeks period [14]. Compared to our study some of the same issues was addressed in the intervention in the study by Stinson and colleagues; management of pain and negative thoughts. However, the interventions differed in regards to the way the intervention was delivered, as well as the age of the included children.

None of the previous intervention studies on children with arthritis have controlled for differences in disease status [12–14]. The disease status in JIA is not constant and flare in the disease may have an impact on differences in pain levels and other outcome variables.

Even though no effect on pain and disability were found, improvement in both the overall HRQL and the arthritis-specific HRQL measures of worries and communication and in pain cognitions were found in the intervention condition compared to the waitlist condition. Only one previous study focusing on self-management in children with JIA included a HRQL measure; but found no improvements [14]. The present intervention provided strategies and training that focused on reducing worries and improving communication skills. The results indicated that the participants had implemented these strategies and found them useful. The intervention showed an effect on pain cognitions demonstrated by moderate to large improvements in the beliefs in the ability to control pain, self-efficacy and the reduction in pain catastrophizing.

The present study had several limitations that may affect interpretation of the results. First, it was limited by a small sample size. A low sample size is often a problem in pediatric intervention studies [40]. This is partly caused by the low prevalence rates of pediatric disorders including JIA. Second, the study was not randomized. However, considering the small sample size, a successful randomization would be difficult to achieve in this study. Third, for ethical reasons, the participants in the waitlist condition were offered the intervention after the end of the waiting period. Thus the design allowed no interference of possible long-term effects of the treatment. Fourth, the disease activity index has not been validated in a large patient population. However, the disease activity score applied in the present study had been used previously in related studies [9, 33].

## Conclusions

So far limited research has been conducted evaluating the efficacy of psychological therapy for children with JIA despite daily pain experience in a significant amount of the children.

The feasibility of the CBT intervention seemed high with regards to the acceptability of the families participating in the intervention. However, the feasibility was challenged by implementation problems with a low response rate. Changes to the study design will be recommendable for further studies.

No reduction in pain was found even though pain management was the main target. However, increased quality of life and improvements in adaptive pain cognitions was reported in the intervention condition compared compared to the controls, even though the disease activity increased in the treatment condition but not in the control condition. The disease activity may influence the child’s pain experience and affect the efficacy of a psychological intervention. Measuring disease activity as well as controlling for changes is recommendable for further studies within this population.

Conclusions on the strength of the efficacy require further research in a large, randomized controlled trial.
